# Screening the Medicines for Malaria Venture Pathogen Box across Multiple Pathogens Reclassifies Starting Points for Open-Source Drug Discovery

**DOI:** 10.1128/AAC.00379-17

**Published:** 2017-08-24

**Authors:** Sandra Duffy, Melissa L. Sykes, Amy J. Jones, Todd B. Shelper, Moana Simpson, Rebecca Lang, Sally-Ann Poulsen, Brad E. Sleebs, Vicky M. Avery

**Affiliations:** a Discovery Biology, Griffith Institute for Drug Discovery, Griffith University, Nathan, Queensland, Australia; b Compounds Australia, Griffith University, Nathan, Queensland, Australia; c The Walter and Eliza Hall Institute of Medical Research, Parkville, Victoria, Australia; d Department of Medical Biology, The University of Melbourne, Parkville, Victoria, Australia

**Keywords:** high-throughout screening, *in vitro*, Leishmania, MMV Pathogen Box, plasmodium, trypanosoma, drug discovery, open-source drug discovery

## Abstract

Open-access drug discovery provides a substantial resource for diseases primarily affecting the poor and disadvantaged. The open-access Pathogen Box collection is comprised of compounds with demonstrated biological activity against specific pathogenic organisms. The supply of this resource by the Medicines for Malaria Venture has the potential to provide new chemical starting points for a number of tropical and neglected diseases, through repurposing of these compounds for use in drug discovery campaigns for these additional pathogens. We tested the Pathogen Box against kinetoplastid parasites and malaria life cycle stages *in vitro*. Consequently, chemical starting points for malaria, human African trypanosomiasis, Chagas disease, and leishmaniasis drug discovery efforts have been identified. Inclusive of this *in vitro* biological evaluation, outcomes from extensive literature reviews and database searches are provided. This information encompasses commercial availability, literature reference citations, other aliases and ChEMBL number with associated biological activity, where available. The release of this new data for the Pathogen Box collection into the public domain will aid the open-source model of drug discovery. Importantly, this will provide novel chemical starting points for drug discovery and target identification in tropical disease research.

## INTRODUCTION

The face of drug discovery is continually changing and never more so than in the last 5 to 10 years where early-stage drug discovery has transitioned to extend beyond the traditional domain of large pharmaceutical companies. Open drug discovery has evolved as a component of this transition and is a major contributor to combatting diseases primarily affecting the poor through identifying and optimizing potential compounds for a range of pathogens (1–10). The open drug discovery approach can be described by several models as recently defined by Wells et al. (Medicines for Malaria Venture [MMV]) in which malaria drug discovery is discussed in the open arena (11). The success of this approach is not limited to malaria (12–16) and includes a number of diseases, such as tuberculosis (17, 18), schistosomiasis (19–21), diseases caused by kinetoplastids (22), toxoplasmosis (23), and cryptosporidiosis (24); in addition, treatments for diseases caused by other viral and bacterial pathogens have also benefited. The open-access drug discovery model, whereby free access and supply of compounds, such as the MMV Malaria Box and subsequently the MMV Pathogen Box (PBox), is made available to researchers with the condition that data obtained are shared with the wider community, has rapidly taken hold.

The first open-access compound set provided by MMV was the Malaria Box (25). More than 200 research groups were involved in a worldwide collaboration to evaluate these compounds against a diverse array of disease indications, with 236 different *in vitro* screens performed. The outcome of this consolidated effort is the continuation of approximately 30 projects where compounds were demonstrated to exhibit activity for different pathogens, as well as for cancers (26).

Following the success of the Malaria Box, MMV has provided the PBox (http://www.pathogenbox.org/) as a second open-access compound collection. The 376 compounds selected for the PBox demonstrate activity against a range of different pathogens, predominantly Plasmodium, Mycobacterium, and kinetoplastid parasites (Trypanosoma brucei, Leishmania spp., and Trypanosoma cruzi). In addition, a smaller number of compounds was selected with activity against a range of other pathogens, including Schistosoma, Toxoplasma, Cryptosporidium, helminths, and dengue. Also included within the PBox is a set of 26 reference compounds with activity associated with one or many pathogens. As part of the PBox compound package, MMV has provided the biological activity of compounds from allied screening platforms (ChEMBL-NTD [https://www.ebi.ac.uk/chemblntd]), along with the plate layout and compound details (structure, trivial name, salt form, and cLogP) in the form of an Excel spreadsheet, referred to here as the PBox supporting information.

The first successes from PBox have recently been published with the identification of Tolfenpyrad (MMV688934), a pyrazole-5-carboxamide based insecticide with activity against the helminth, barber's pole worm (27). Second, the demonstration of inhibitory activity against Candida albicans biofilm formation by MMV688768 (an original indication for schistosomiasis) (28).

Our extensive well-established and validated image-based assays for neglected tropical disease drug discovery provided the ideal platform to enable biological profiling and direct comparative analysis of the PBox against a number of pathogens simultaneously. In close collaboration with both MMV and the Drugs for Neglected Diseases Initiative, innovative image-based assays for the following parasites and life cycle stages have been established. Plasmodium falciparum asexual blood stage (ABS) (29) and late-stage gametocyte (LSG) (14) assays supporting numerous hit identification, hit-to-lead, and lead optimization projects have been developed. The development of a 384-well surrogate assay for human African trypanosomiasis (HAT), which utilizes the parasite *Trypanosoma brucei brucei* (30), was the first of its kind to be published. Image-based assays for both Trypanosoma cruzi (31) and Leishmania donovani provide essential support for compound profiling for hit-to-lead and lead optimization, contributing valuable insights into mechanism of action of these lead molecules. The P. falciparum, *T. brucei brucei*, and T. cruzi assays are well validated and have been used extensively for a number of years in the identification and characterization of compounds active for these particular pathogens (31–47).

The PBox compounds are each provided as 10 μl of 10 mM dimethyl sulfoxide (DMSO) stock solutions. In order to maximize this resource and ensure reproducible, high-quality data, liquid handling and compound management expertise, in combination with well-validated highly reproducible assay formats, is required. Compounds Australia (48) is a world class compound storage and handling facility, providing compound management research logistics within Australia and internationally to support diverse open and closed drug discovery programs. This enabling infrastructure and expertise allows for small-volume compound handling and therefore the ability to test not only the dose response at concentrations higher than 1 μM (as suggested by MMV) but also against multiple parasites from an initial 10-μl volume.

The compounds within PBox are categorized into disease indication subsets by MMV and partners, where compounds demonstrated a minimum of 5-fold selectivity for the pathogen over mammalian cells (www.pathogenbox.org/about-pathogen-box/supporting-information). Excluding the reference set, 33% of the PBox collection is comprised of compounds with activity against Plasmodium, with another 30% active against tuberculosis and 18% active against kinetoplastid species. The remaining 19% of the compounds are distributed through the other pathogen disease indications. Rather than testing only compounds with no antiplasmodial or antikinetoplastid activity, as defined by the PBox supporting information, all compounds were tested in multiple-parasite assays in order to provide comparative data from a second screening platform versus that originally used to generate the initial PBox biological data. The assays consisted of four high-content-imaging platforms, namely, P. falciparum asexual blood stage (ABS) and late-stage gametocytes (LSG), plus the intracellular forms of the kinetoplastid pathogens T. cruzi and L. donovani, as well as a viability assay for *T. brucei brucei* utilizing the fluorescent metabolic indicator resazurin.

In order to thoroughly assess the suitability of selectively active compounds as potential chemical starting points for hit-to-lead, lead optimization, or target identification programs, an extensive review of the scientific literature and activity databases was performed and collated with the biological activity data we generated. This combined information has provided the means to select compounds both within their respective disease indication set (especially within the kinetoplastid set) and also within those of other disease indications. By both confirming and identifying potential chemical starting points for drug discovery programs for malaria, HAT, Chagas disease, and leishmaniasis and the publishing of the data generated in free access databases, the open-source model will be greatly advanced by the availability of these data.

We believe that the activity data generated, the information gathered, and the indication of new chemical starting points will ultimately facilitate more successful open-source drug discovery programs fueling the identification of new drug candidates for neglected and tropical diseases worldwide.

## RESULTS

The complete screening data set for all five assays is presented in Supplemental File 1, with the data represented as an activity heat map. The details for all compounds, excluding the reference compound set from the PBox supporting information Excel file, are aligned and merged with the biological activity obtained from the evaluation undertaken within Discovery Biology. This in turn was aligned with the biological activity of the kinetoplastid, Plasmodium, and HepG2 activity data provided within the PBox supporting information file.

The primary screening data for each Discovery Biology assay was used to color code activity and subsequently applied to both PBox data and Discovery Biology IC_50_s (see Supplemental File 1; the second tab contains the color code for the activity heat map). Evaluation of the compounds for their suitability for repurposing or for progression into hit-to-lead or target identification studies was performed using searches on ChEMBL and PubMed, along with SciFinder and Chemspider. All information gathered, including literature citations, alternative aliases, ChEMBL numbers, ChEMBL biological activity data (in the case of malaria), and commercial availabilities (Table 1) is incorporated into Dataset S1 in the supplemental material.

**TABLE 1:** Percentages of compounds for each pathogen for which a commercial supply of the compound was identified

Pathogen set	No. of compounds		% with a commercial supplier
	In PBox	Commercial supplier identified	
Cryptosporidiosis	13	2	13
Dengue	5	0	0
Hookworm	1	1	100
Kinetoplastids	70	25	36
Lymphatic filariasis	3	1	34
Malaria	125	84	67
Onchocerciasis	11	3	27
Schistosomiasis	13	9	69
Toxoplasmosis	15	2	13
Trichuriasis	1	0	0
Tuberculosis	116	83	71
Wolbachia LF	3	0	0

### Commercial supplier.

Using the SMILE string from the PBox information file, we performed as search for chemical suppliers for all of the compounds within PBox, primarily with Chemspider and SciFinder (http://www.cas.org/products/scifinder). Supplier availability is presented as a percentage of the total number of compounds per disease set (Table 1).

### Reference compound set.

The PBox contains 26 compounds used as internal references for evaluations. The reference compound set was tested directly in an 11-point dose-response format used to calculate the 50% inhibitory concentrations (IC_50_s), as described in Supplemental File 2. The data are presented in Table 2. The compounds were tested twice in the L. donovani assay due to insolubility problems with certain active compounds, such as amphotericin B, miltefosine, and buparvaquone. The activities of many of these compounds against Plasmodium and kinetoplastid parasites have been published previously (49, 50).

**TABLE 2:** Reference compound IC_50_ data

Compound	IC_50_ (μM)^*a*^								
	P. falciparum		Mammalian cell cytotoxicity	T. cruzi			*T. brucei brucei*	L. donovani	
	3D7 ABS	NF54 LSG	HEK293	Intracellular amastigotes	3T3 host cells	*E*_max_	Trypomastigotes	*n* = 1	*n* = 2
Doxycycline	90%	77%		IA	IA		IA	IA	IA
Mefloquine	<0.016	5.61		4.7	IA	99	0.33	5.17	6.58
Primaquine	91%	96%		94%	73%		91%	33%	79%
Pentamidine	0.01	1.43	75%	IA	IA		<0.02^D^	IA	IA
Sitamaquine	2.03	0.982	100%	IA	IA		91%	IA	IA
Nifurtimox	IA	39%		1.42^A^	IA	100	5.38	IA	IA
α-Difluoromethylornithine	IA	IA		IA	IA		IA	IA	IA
Praziquantel	IA	IA		IA	IA		IA	IA	IA
Diethylcarbamazine	IA	IA		IA	IA		IA	IA	IA
Mebendazole	IA	IA		IA	IA		IA	IA	IA
Suramin	IA	IA		IA	IA		0.11	IA	IA
Amikacin	IA	IA		IA	IA		IA	IA	IA
Levofloxacin (–)-ofloxacin	IA	IA		IA	IA		IA	IA	IA
Clofazimine	84%	IA		IA	IA		IA	IA	IA
Ethambutol	IA	42%		IA	IA		IA	IA	IA
Linezolid	IA	IA		IA	IA		IA	IA	IA
Benznidazole	IA	IA		94%	IA		IA	IA	IA
Posaconazole	4.1	63%		0.172^B^	IA	73	IA	64%	60%
Rifampin	2.23	78%	IA	IA	IA		IA	IA	IA
Auranofin	0.70	0.55	2.20	3.02	4.90		0.24	0.63	2.00
Miltefosine	IA	IA		IA	IA		IA	11.69	2.26
Nitazoxanide	IA	IA		IA	IA		41%	44%	41%
Streptomycin	IA	41%		IA	IA		IA	IA	IA
Amphotericin B	87%	IA		IA	IA		IA	100%^C^	IA^C^
Buparvaquone	0.14	5.3	IA	4.92	IA	98.5	IA	1.88	60%
Bedaquiline	91%	75%		IA	IA		59%	IA	IA

a The activities of all 26 compounds are presented for all assays plus HEK cytotoxicity. IA, inactive at the top screening dose (20 μM for *P. falciparum*, *L. donovani*, and *T. brucei brucei*; 16.4 μM for T. cruzi). Percentage values, where specified in the body of the table, indicate the percent inhibition at the top screening dose (20 μM for P. falciparum, L. donovani, and *T. brucei brucei*; 16.4 μM for T. cruzi). Standard in-house assay controls are indicated by superscript capital letters: A, 1.23 μM; B, 0.001 μM; C, 0.41 and 0.30 μM; D, <0.001 μM. *E*_max_ is the maximal % inhibition plateau generated in compound dose response curve analysis.

Whereas one of the replicates for amphotericin B contained within the PBox compound set was not active against L. donovani when tested at 20 μM, the amphotericin used as an in-house control in the same assay was active, as expected. Given the poor solubility of this compound in water, which was used to generate an intermediate dilution plate, it is possible that at high concentrations the compound did not solubilize completely. Pentamidine received in the PBox compound collection also did not show activity at 20 μM against L. donovani. It is reported that this drug reduces the number of parasites per macrophage in *in vitro* experiments but not the number of infected macrophages as assessed here (51).

### Evaluation of individual parasite selectivity for the kinetoplastid disease indication compound set.

**FIG 1:**
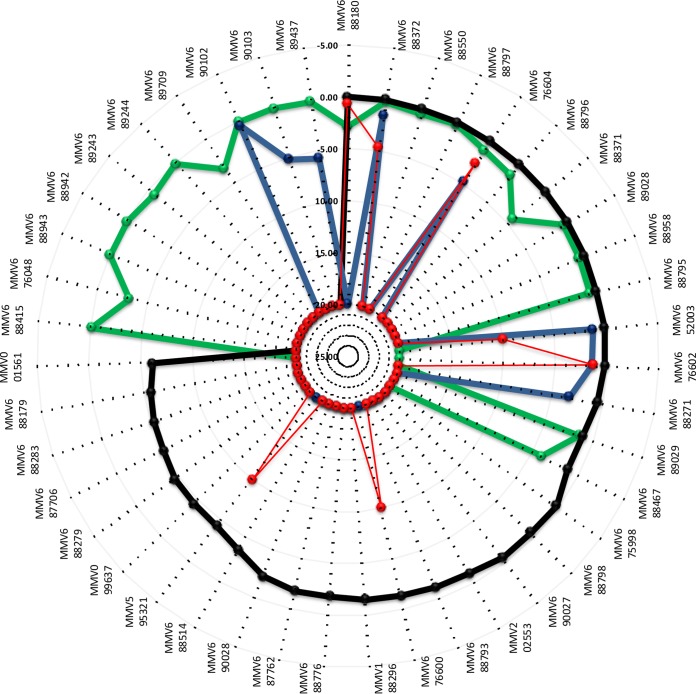
Radar activity plot of IC_50_s (μM) for MMV Pathogen Box compounds: *T. brucei brucei* (black), T. cruzi (green), L. donovani (blue), and HEK293 mammalian cell cytotoxicity (red). The activity scale is IC_50_s in μM.

Seventy compounds within the PBox have demonstrated published activity against L. donovani, *T. brucei brucei*, and/or T. cruzi or various dually active combinations and are clustered within the single kinetoplastid set within the PBox supporting information. A large number of these compounds had no cytotoxicity data provided within the PBox supporting information. In order to classify compounds as individually parasite selective, all compounds demonstrating activity within the Discovery Biology kinetoplastid assays, namely, 43 of the 70, were also evaluated for cytotoxicity using the HEK293 cell line. Selectivity of >5-fold (as used by MMV) for the parasite over HEK293 cytotoxicity was used to determine whether compound activity was parasite selective. A summary of the activity data is provided in Fig. 1.

Three-quarters of the kinetoplastid compounds identified as active by the Discovery Biology assays were active against *T. brucei brucei*, with approximately one-quarter also active against T. cruzi and only approximately one-fifth active against L. donovani. Three of the eight compounds that displayed activity against L. donovani also exhibited similar activity in the HEK293 cytotoxicity assay and were therefore classified as not selective.

### Comparison between Discovery Biology and PBox data sets for kinetoplastid and Plasmodium disease indication compounds.

**FIG 2:**
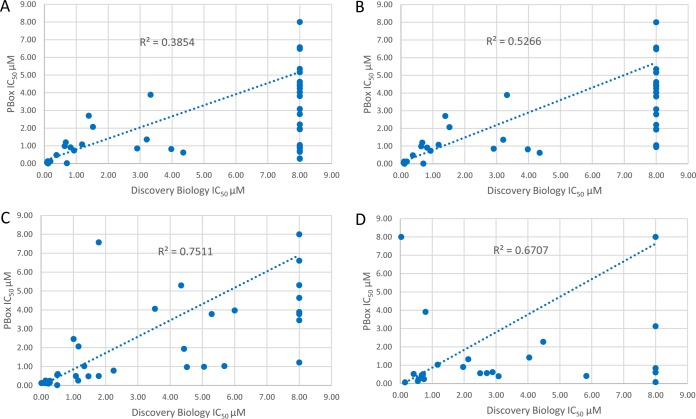
Correlation of Discovery Biology data for compounds tested against T. cruzi, *T. brucei brucei*, and P. falciparum gametocyte versus respective PBox activity data provided. (A) *T cruzi* (all compounds); (B) T. cruzi (5 discrepant compounds were removed); (C) *T. brucei brucei*; (D) P. falciparum gametocytes.

To accommodate the screening of all pathogens from the same compound set provided, 5 mM concentrations of compound stocks were prepared. Thus, due to compound limitations, IC_50_s greater than 8 μM could not be determined since the maxiumum effect (*E*_max_) values were not obtainable. To perform a comparison with the PBox activity data, all compounds with activity greater than 8 μM, for either assay, were assigned an IC_50_ of 8 μM in order to perform comparisons of the compounds within biologically relevant levels of activity. These data are presented in Fig. 2.

Of the 70 compounds within the kinetoplastid pathogen set, 65 were compared for T. cruzi activity, and the results are presented in Fig. 2A. Five compounds from the PBox were not tested against T. cruzi in the supporting information file; thus, a comparison could not be made. The activity data comparison for all 65 compounds has an *r*^2^ value of 0.385, which is low. However, with the removal of five compounds (MMV652006, MMV688271, MMV688776, MMV688179, and MMV658988) that had no activity at 8 μM in the Discovery Biology assay and <1 μM in the PBox supporting information file, the *r*^2^ value increased to 0.527. It would appear that a small number of compounds have different activities between the two assays but that, in general, activity against T. cruzi correlated well with the PBox data (Fig. 2A and B). There were 25 compounds (30%) with demonstrated IC_50_s against T. cruzi (as high as 25 μM IC_50_s) in the PBox supporting information file (undertaken at the Laboratory of Microbiology, Parasitology, and Hygiene [LMPH], University of Antwerp) for which an IC_50_ could not be determined. Eleven of these compounds displayed >50% inhibitory activity at 18.3 μM (top dose tested); however, none showed >50% activity at 9.15 μM (second dose), suggesting theoretical IC_50_s higher than 8 to 9 μM.

The variance in the activity of these compounds may be attributed to differences in the assay formats. The LMPH assay utilized a C4 β-galactosidase reporter gene-transfected parasite in human lung fibroblast (MRC 5SV2) host cells exposed to compound for 7 days compared to 2 days of exposure for the Discovery Biology assay using 3T3 host cells infected with nontransfected parasites. It is possible that these compounds are either slow acting and therefore not detected in the shorter 48-h exposure assay or the transgenic parasite and MRC5SV2 host cells were contributing factors to differences in compound activity. Also, without details of any corresponding effects on the host cells by the compounds, it is impossible to determine whether parasite selectivity over the MRC5SV2 host cells was impacted upon and thus would be more evident during the longer incubation period.

A correlation of Discovery Biology *T. brucei brucei* versus PBox *T. brucei brucei* is presented in Fig. 2C. Sixty-four of the compounds within the kinetoplastid set were available for comparison. There was a high degree of correlation (*r*^2^ = 0.751) between the Discovery Biology *T. brucei brucei* activity compared to PBox (Fig. 2C), particularly for compounds that possess potent activity (IC_50_ < 2 μM), but a higher degree of deviation was observed for compounds with IC_50_s of >4 μM.

The PBox contains 125 compounds with antiplasmodial activity. For 91% of these compounds, the P. falciparum ABS and LSG assays from Discovery Biology and the data contained within the PBox supporting information Excel file aligned well (*r*^2 =^ 0.67 for LSG). The ABS correlation between the two screening platforms cannot be made for IC_50_s, since these compounds were only taken for IC_50_ evaluation if the degree of activity at primary testing was considerably different from that provided in the PBox supporting information file. A high degree of correlation was observed between assays with most of the compounds.

A correlation of the Discovery Biology stage IV/V gametocyte assay with the PBox stage V gametocyte assay is presented in Fig. 2D. One hundred and twenty compounds from the 125 malaria compound set were compared. The correlation of activity between the Discovery Biology LSG assay (stage IV) to that of stage V gametocyte data (saponin-lysis sexual stage assay [SaLSSA]) (52) within the PBOX supporting information file indicates good correlation between these two assays (*r*^2^ = 0.67). A high level of correlation for the majority of compounds was observed, though five had differing levels of activity between the two assays (MMV023233, MMV023985, MMV1019989, MMV1029203, and MMV1037162). In general, the PBox stage V assay showed higher activity for these compounds than those determined by the Discovery Biology stage IV imaging assay when we tested the compounds the MMV PBox supplied. Seven of the compounds present within PBox with low ABS activity and greater activity against stage V gametocytes (MMV062221, MMV1019989, MMV1029203, MMV1030799, MMV1037162, and MMV1088520) had been previously identified from an HTS campaign of a malaria-naive library of 80,000 compounds performed by Discovery Biology (53). On testing freshly solubilized stocks of MMV1019989 (diaminotriazine), MMV1037162, and MMV1029203 (pyrdiyl thienopyrimidine) it has been noted (S. Duffy, unpublished observations) that the compound-treated assay wells had a glassy and/or shiny appearance and, under light microscopy, red blood cell (RBC) lysis was witnessed in the ABS assay and, to a lesser extent, the gametocyte assays at screening concentrations of ≥2 μM. Images of artemisinin-treated wells compared to MMV1037162-treated wells are presented in Supplemental File 2, which shows the destruction of the RBCs at the end of the 72-h assay incubation. The activity of these three compounds was found to be highly variable upon repeat testing by the Discovery Biology imaging assays (Duffy, unpublished).

MMV009135 has no activity data within the PBox supporting information file. This compound is the regioisomer of the compound initially identified as active (54, 55) and did not show any activity within the Discovery Biology antiplasmodial assays. MMV016838 also has no activity data within the PBox supporting information file, but the activity is indicated as “known to be active” (ktba) against both gametocytes and liver-stage *in vitro* assays. However, this compound was found not to be active in either the Discovery Biology ABS or gametocyte assays. MMV676270 also demonstrated no activity against either the ABS or LSG Discovery Biology assays in comparison to literature ABS activity data (0.99 μM) (54). The PBox supporting information file has no activity provided for direct comparison of this batch of compound supplied. MMV011229, according to the PBox supporting information, is ABS active (0.38 μM) and reported as “ktba” for gametocytes and the liver stage. However, although the ABS data are in agreement, the stage IV Discovery Biology gametocyte assay did not find this compound to be active. MMV006901 had ABS activity confirmed with that from the PBox supporting information, but the Discovery Biology gametocyte assay found this compound to be only moderately active against stage IV gametocytes at primary testing, and thus an IC_50_ was not determined. Nine compounds (MMV007133, MMV007625, MMV007920, MMV019742, MMV020291, MMV02388, MMV676358, MMV676442, and MMV687794) demonstrated variable LSG activity to that contained within the PBox supporting information file. Overall, however, the Plasmodium activity data were generally consistent between Discovery Biology and the PBox.

It should also be noted that aspects other than assay technology come into play in these multicenter compound screening approaches. Assay medium composition and protein binding of the compounds may also affect the activity of some compounds more than others (25). Compound solubility, stability in DMSO solution over time, plate storage conditions, and dilution protocols can all contribute to variances in activities between screening platforms and between laboratories.

### New and novel parasite biological activity for potential repurposing of compounds.

Table 3 lists 13 compounds from the other disease indication sets with antikinetoplastid activity where biological activity is new to the PBox activity data.

**TABLE 3:** Compounds from other disease sets with antikinetoplastid activity

Compound ID	ChEMBL DB reference	Disease set within pathogen box	New selective indication (PBox)	IC_50_ (μM) for new indication	Compound class	Predicted cellular target of associated class with new disease indication	New disease reference	Mammalian cellular target of associated class	Reference for mammalian cellular target of associated class
MMV687776		Lymphatic filariasis	T. cruzi	2.39	Benzoxaborole warhead	No hits	57, 58		
MMV022478	54	Malaria	T. brucei brucei	1.45	Pyrazolo[1,5-a]pyrimidine	No hits	No hits	NADPH oxidase 4	56
MMV022029	54	Malaria	T. brucei brucei	2.38	Biaryl sulfonamide	No hits	No hits	No hits	
MMV028694	54	Malaria	T. brucei brucei	2.39	2,4-Disbustituted pyrimidine	No hits	No hits	No hits	
MMV006901	54, 55	Malaria	T. brucei brucei	3.36	2,4-Aminoquinoline	No hits	No hits	No hits	
MMV010576	54	Malaria	L. donovani	5.18	2-Amino-3,5-diaryl pyridine	No hits	No hits	Kinase	Numerous
MMV688768		Schistosomiasis	T. brucei brucei	1.50	2,3-Disubstituted Indole	No hits	No hits	Alanyl aminopeptidase and dipeptidyl peptidase	61
MMV688417	68	Toxoplasmosis	T. cruzi	1.43	Pyrazolo[3,4-d]pyrimidin-4-amine	Kinase	64	PI3K/AKT/mTOR pathway	Numerous
MMV637229		Trichuriasis	T. cruzi	1.22	Diarylmethane ether	No hits	No hits	Antihistamine	62
MMV687248	18	Tuberculosis	T. brucei brucei, T. cruzi	1.05, 4.00	3,5-Disubstituted pyridine	No hits	No hits	No hits	
MMV687273	69–73	Tuberculosis	T. brucei brucei, L. donovani	2.28, 3.47	Acyclic monoterpene	HMG-CoA reductase pathway?	65	No hits	
MMV153413		Tuberculosis	T. brucei brucei	2.99	Tetrasubstituted thiophene	No hits	No hits	SIRT2	61
MMV021013	18	Tuberculosis	T. cruzi, L. donovani	1.67, 1.48	2-Pyridyl-4-aminopyrimidine	Methionine aminopeptidase	66, 67	Methionine aminopeptidase; nuclear factor κB ligand (RANKL)	62, 63

Table 4 lists the 31 compounds identified from screening the PBox against the Discovery Biology antiplasmodial assays which are from other disease indication compound sets. The activity identified is new to the PBox data set and, after chemical class identification and literature review, 10 compounds were identified as having novel activity against P. falciparum.

**TABLE 4:** Compounds from “other disease indication” sets demonstrating antiplasmodial activity

Compound ID	Disease set within PBox	Antiplasmodial IC_50_ (μM)	Compound class	Predicted cellular target of associated class with new disease indication	New disease reference	Mammalian cellular target of associated class	Reference for mammalian cellular target of associated class	Novel antimalarial activity
MMV675968	Cryptosporidiosis	0.03 (ABS)	Quinazoline-2,4-diamine	Dihydrofolate reductase	76	Folate pathway	74	No
MMV688547	Kinetoplastids	1.02 (ABS)	Bisarylamidine	DNA-targeted agent; DNA minor groove binding at AT-rich DNA sequences	77	No hits		No
MMV688362	Kinetoplastids	0.59 (ABS)	Bisarylamidine	As above	77	No hits		No
MMV688179	Kinetoplastids	0.59 (ABS)	Bisarylguanidinium	As above	77	No hits		No
MMV688271	Kinetoplastids	1.21 (ABS)	Bisarylguanidinium	As above	77	No hits		No
MMV687749	Tuberculosis	2.08 (ABS)	2-Amino-4-aryl ether pyrimdine	No hits	No hits	Burtons tyrosine kinase	75	Yes
MMV687765	Tuberculosis	2.01 (ABS)	2-Amino-4-aryl ether pyrimdine	No hits	No hits	Burtons tyrosine kinase	75	Yes
MMV659004	Kinetoplastids	1.86 (ABS)	2-Pyridyl-4-aminopyrimidine	Methionine aminopeptidase	66, 67	Methionine aminopeptidase; nuclear factor kappa-B ligand (RANKL)	62, 63	No
MMV658988	Kinetoplastids	2.18 (ABS)	2-Pyridyl-4-aminopyrimidine	As above	66, 67	Methionine aminopeptidase; nuclear factor κB ligand (RANKL)	62, 63	No
MMV659010	Kinetoplastids	1.71 (GAM)	2-Pyridyl-4-aminopyrimidine	Methionine aminopeptidase	66, 67	Methionine aminopeptidase; nuclear factor κB ligand (RANKL)	62, 63	No
MMV688122	Tuberculosis	2.11 (ABS), 0.11 (GAM)	2- Pyridyl thieno[3,2]pyrimidine	Methionine aminopeptidase?	66, 67	Methionine aminopeptidase; Receptor activator of nuclear factor κB ligand (RANKL)?	62, 63	No
MMV661713	Tuberculosis	1.49 (ABS)	4-Pyridyl-2-aryl pyrimidine	No hits	No hits	Glutaminyl cyclase	78	Yes
MMV652003	Kinetoplastids	0.92 (ABS)	Benzoxaborole warhead	Leucyl-tRNA synthetase	79	No hits		No
MMV021057	Malaria (GAM)	0.03 (GAM)	β-Methoxyacrylate analogue (azoxystrobin, a known fungicide)	*bc*_1_ complex(?)	80	No hits		No
MMV688754	Kinetoplastids (GAM)	0.14 (GAM)	Oxime analogue (trifloxystrobin, a known fungicide)	No hits	80	No hits		No
MMV671636	Onchocerciasis	1.01 (ABS)	Quinolone	Mitochondrial cytochrome *bc*_1_ complex	36	No hits		No
MMV688279	Kinetoplastids	0.32 (ABS), 2.35 (GAM)	Dihydroquinazoline	No hits	81, 82	No hits		Yes/no?
MMV687703	Tuberculosis	1.33 (ABS), 3.83 (GAM)	2-Aryl imidazole	No hits	No hits	No hits		Yes
MMV688550	Kinetoplastids	2.48 (ABS)	Imidazo[1,2]purine	No hits	No hits	No hits		Yes
MMV688283	Kinetoplastids	0.66 (ABS), 4.10 (GAM)	4-Amino quinoline	No hits β-hematin?	86	No hits		No
MMV676383	Tuberculosis	4.43 (ABS), 1.37 (GAM)	2-Substituted benzooxazole	No hits	No hits	No hits		Yes
MMV688703	Toxoplasmosis	3.16 (ABS)	Trisubstituted pyrrole	cGMP-dependent protein kinase	87, 88	p38 kinase	83	No
MMV688766	Schistosomiasis	0.85 (ABS), 1.31 (GAM)	Trisubstituted isooxazole	No hits	No hits	PPAR agonist (?)	84	Yes
MMV676388	Tuberculosis	4.93 (ABS), 2.99 (GAM)	5-Sulfonyl tetrazole	No hits	No hits	Thioredoxin reductase	85	Yes
MMV023969	Tuberculosis	0.59 (ABS)	Isoquinoline	No hits	No hits	No hits		No*
MMV024311	Tuberculosis	0.75 (ABS)	2,3-Disubstituted indole	No hits	No hits	No hits		No*
MMV021660	Tuberculosis	0.16 (ABS)	2,4-Diamino pyrimidine	Folate pathway	81, 89–92	Too numerous		No

Twelve compounds from the kinetoplastid disease indication set were identified, with two demonstrating novel antiplasmodial activity: MMV688550 and MMV68827. The tuberculosis disease indication set resulted in the greatest number of novel antiplasmodial active compounds. From 14 compounds identified from the tuberculosis compound set, 7 were deemed as novel for activity against P. falciparum (MMV661713, MMV687703, MMV687273, MMV687749, and MMV687765 having activity less than 2 μM, with MMV676383 and MMV676388 demonstrating only moderate activity between 4 and 5 μM). Although one compound each was identified from four other disease indication sets (cryptosporidiosis, schistosomiasis, toxoplasmosis, and onchocerciasis), only one schistosomiasis compound (MMV688766) demonstrated novel antiplasmodial activity. However, a number of compounds, although not novel in their antiplasmodial activity, do not have a known drug target and therefore provide interesting starting points for target elucidation and validation studies.

## DISCUSSION

The PBox presents a significant opportunity for identification of compounds for progression into hit-to-lead and target identification projects for a variety of pathogens. Detailed information regarding the compound suitability for repurposing or for taking forward into hit-to-lead or target identification studies has been gathered. Searches were performed using ChEMBL and Scifinder, and the information gathered was collated, including the kinetoplastid and Plasmodium supporting information from PBox. All the literature search information was incorporated into supporting information (see Dataset S1 in the supplemental material). The third worksheet within the Excel file contains the citations for the pathogen activity present in ChEMBL.

All the assays performed in this evaluation demonstrated good to reasonable (*r*^2^ = 0.52 to 0.75) correlations with the data within the PBox supporting information; however, some compounds displayed differences in the activity/inactivity status or the level of activity obtained. The complete set of activity data is presented in the form of a heat map, including IC_50_s and the level of activity (a heat map color code is presented in Dataset S1 in the supplemental material). This information and alignment of data for the compounds against Plasmodium and the kinetoplastids (*T. brucei brucei*. L. donovani, and T. cruzi) is a valuable tool for researchers in the field of drug discovery.

Twenty-six reference compounds were also included in PBox, although activity data are not provided within the PBox supporting literature. The data provided here are therefore a valuable reference tool for determining variances within assay performance between laboratories. For projects run across numerous laboratories, it is essential to be able to compare the activity of reference compound sets to monitor assay performance. The activity data for the references are presented in Table 2.

### Potential chemical starting points for drug discovery. (i) New and novel compounds identified against kinetoplastids.

Of the compounds not within the Pbox defined kinetoplastid set, 21 were identified with activity against L. donovani, T. cruzi, or *T. brucei brucei*. However, if the selectivity criteria of >5-fold (as used by MMV for inclusion within PBox) over either HEK293 or HEPG2 is applied, only 13 (57%) of the compounds identified by the Discovery Biology assays displayed selective, antiparasitic activity (Table 3).

Five compounds from the malaria disease indication set were identified as selective: four for *T. brucei brucei* (MMV022478, MMV022029, MMV028694, and MMV006901, IC_50_s of 1.45 to 3.36 μM) and one for L. donovani (MMV010576, IC_50_ of 5.18 μM). The activity for these compounds is novel for *T. brucei brucei*. A literature search revealed that these active compounds were not associated with a mechanism of action apart from a potential link to trypanothionine reductase for MMV022478. The pyrazolo[1,5-*a*]pyrimidine class, to which MMV022478 belongs, has been reported to inhibit mammalian NADPH oxidase 4 (56). In *T. brucei brucei* it has been proposed that the enzyme trypanothione reductase (TryR) may function as an NADPH oxidase (57). TryR is essential and a potential drug target in *T. brucei brucei* (58, 59). However, despite the numerous inhibitors that have been designed to target TryR, none have progressed past *in vitro* studies (60–62).

The tuberculosis PBox subset yielded 10 compounds with activity against kinetoplastids; however, only 4 compounds (MMV687248, MMV687273, MMV021013, and MMV153413) displayed >5-fold selectivity over mammalian cell cytotoxicity. MMV687273, the clinical candidate SQ-109, was active against *T. brucei brucei*, T. cruzi, and L. donovani, as well as P. falciparum. Based on the HEK293 activity, the compound was selective for all four parasites but only showed a marginal window with respect to HEPG2 activity. SQ-109 has been extensively studied for T. cruzi, *T. brucei brucei*, and L. donovani; therefore, further studies on this compound may be of limited benefit (63, 64). MMV687248, a 3,5-disubstituted pyridine with an IC_50_ of 1.05 μM against *T. brucei brucei* with a selectivity index (SI) of >20 against both HEK293 and HEPG2, is an interesting novel starting point for HAT drug discovery. MMV021013, a 2-pyridyl-4-aminopyrimidine, was active against all three parasites but demonstrates greater activity against T. cruzi (1.67 μM) and L. donovani (1.48 μM) than against *T. brucei brucei* (3.51 μM). Activity against Plasmodium and Leishmania has been reported (65, 66); however, activity against T. cruzi has not been recorded within the scientific literature. Based on chemical structure, the cellular target of these compounds in Plasmodium is proposed to be methionine aminopeptidase. Therefore, these compounds could also target peptidases in T. cruzi, although this could need to be determined. MMV153413, a tetrasubstituted thiophene, demonstrated moderate activity against *T. brucei brucei* (2.99 μM) and, interestingly, against P. falciparum stage IV gametocytes but not ABS. The activity of this compound in mammalian cells has been related to SIRT2 [(NAD)-dependent deacetylase], which functions as an intracellular regulatory protein (67).

One compound from the schistosomiasis compound set, MMV688768, a 2,3-disubstituted indole, displayed activity against *T. brucei brucei* with an IC_50_ of 1.5 μM and SIs of >13 and >6, respectively, for HEK293 and HEPG2 cells. This compound has demonstrated dual activity against both alanyl aminopeptidase and dipeptidyl peptidase in mammals, with patent literature suggesting a range of medical indications, including autoimmune disease, allergies, and psoriasis (68). A number of dipeptidases have been shown to be essential virulence factors in *T. brucei brucei* infections and thus potential drug targets (69, 70). Inhibitors of *T. brucei brucei* serine peptidases have been identified, and the currently used antitrypanosomal drugs suramin, pentamidine, and diminazene all inhibit *T. brucei brucei* oligopeptidase B, although it is not clear whether this accounts for the antitrypanosomal activity of these drugs (69). Since MMV688768 has low micromolar (1.5 μM) activity and good selectivity against *T. brucei brucei*, further studies with this compound are warranted to determine the target and whether activity is retained against the human infective subspecies.

One compound from the lymphatic filariasis set, MMV687776 (which possesses a benzoxaborole warhead), and one compound from the toxoplasmosis set, MMV688417 (pyrazolo[3,4-d]pyrimidin-4-amine), displayed T. cruzi selectivity in relation to HEK293 cytotoxicity, with IC_50_s of 2.39 and 1.43 μM, respectively. MMV688417 was developed as a calcium-dependent protein kinase inhibitor against T. gondii (72). However, this compound displayed subefficacious activity against T. cruzi amastigotes in the Discovery Biology assay, which suggests a slow-acting mode of action against the parasite. MMV687776 was solely active against T. cruzi based on the testing undertaken in these studies. This compound did not display activity against HEK293; however, based on the PBox data, it did show activity against HEPG2 cells, with an IC_50_ of 10.9 μM and an SI of only 4.3 compared to >8-fold for HEK293 cytotoxicity. Although the activity of benzoxaboroles against trypanosomes and plasmodia has been cited (73–75), their activity solely against T. cruzi is of interest from a target and chemical starting point for hit-to-lead purposes. MMV637229, a diarylmethane ether (clemastine), is active against T. cruzi (1.22 μM) and P. falciparum ABS (2.2 μM). However, the activity of clemastine against T. cruzi, particularly with respect to *in vitro* and *in vivo* drug combinations, has been published (50).

### (ii) New and novel compounds identified against Plasmodium.

When comparing the activity data obtained from Discovery Biology with the PBox supporting information activity data, a number of compounds demonstrated potent antiplasmodial activity not represented within PBox supporting information file. Upon searching for activity within ChEMBL, it was observed that many of the active compounds have already demonstrated antiplasmodial activity. A number of these compounds are not reported within the PBox supporting information file to have antiplasmodial activity, and these findings are therefore considered new data. A number of the compounds from pathogen disease indication sets, including, cryptosporidiosis, kinetoplastids, onchocerciasis, schistosomiasis, toxoplasmosis, trichuriasis, and tuberculosis, have demonstrated new antiplasmodial data for the PBox. These compounds are presented in Table 4.

After clustering by compound class and database searches for antiplasmodial activity, the majority of the compound classes are known to possess activity against P. falciparum. After filtering out known antimalarial actives, several compounds were identified that were not previously known to possess antimalarial activity. Some of these compounds displayed modest activity against ABS P. falciparum parasites, such as MMV687749 and MMV687765, both with the 2-amino-4-aryl ether pyrimidine chemotype from the tuberculosis set, with an IC_50_ of 2.0 μM. The most potent ABS compounds were related to known fungicides used in agriculture. MMV021057, a β-methoxyacrylate analogue of azoxystrobin present in the malaria set, and MMV688754, an oxime analogue of trifloxystrobin from the kinetoplastid set, had IC_50_s of 0.04 and 0.19 μM, respectively. Although the ABS data are not novel for the parent compounds, the identification of potent activity against stage IV gametocytes (0.03 and 0.14 μM, respectively) is novel data. However, determination of whether the parent compounds or their potential metabolites are the active components needs to be undertaken to ascertain whether these compounds are to be taken forward for further optimization (76). Other compounds with potent ABS antimalarial activity—MMV688279, a dihydroquinazoline, and MMV021660, a 2,4-diamino pyrimidine (with IC_50_s of 0.320 and 0.160 μM) from the kinetoplastid and tuberculosis sets, respectively—are more promising from an optimization viewpoint. MMV688279 also possesses modest LSG activity (IC_50_ of 2.4 μM).

Of note is the activity of MMV688547, MMV688362 (bisarylamidine), MMV688179, and MMV688271 (bisarylguanidinium) compounds, which have been identified as binding to the DNA minor groove at AT-rich regions of DNA (77–79). Bisarylamidine and bisarylguanidinium both demonstrate activity against P. falciparum 3D7, but the bisarylamidine compounds in the kinetoplast collection do not have antikinetoplastid activity against the parasites tested either from the PBox supporting information file or the Discovery Biology data, apart from T. brucei rhodesiense. Compounds of this chemotype, but not the specific compound itself, have been reported to have antitrypanosomal activity (79). The bisarylguanidinium compounds MMV688179 and MMV688271 both demonstrate activity for all pathogens within the kinetoplastid set, as represented by the PBox supporting information. Of note is the lack of activity for MMV688179 and MMV688271 within the Discovery Biology T. cruzi assay. It has been mentioned previously that the Discovery Biology T. cruzi assay has a 2-day incubation period compared to the 7-day incubation period for the PBox assay. This could indicate a delayed mechanism of action for this compound. However, additional parameters, such as medium components, compound stability/solubility, or compound processing (such as concentration, diluent, or storage), could contribute to differences observed in compound activity.

Several compounds that possessed P. falciparum ABS activity also possessed LSG activity. MMV659004 (ABS:LSG, 1.86:0.67 μM), MMV658988 (ABS:LSG, 2.18:0.30 μM), MMV021013 (ABS:LSG, 0.46:0.19 μM), MMV688122 (ABS:LSG, 2.11:0.11 μM) and MMV659010 (ABS:LSG, >8:1.71 μM), all from the 2-pyridyl-4-aminopyrimidine class, possessed LSG activities that were ≥2-fold those of the ABS activities. Two other compounds, MMV153413 (1.06 μM) and MMV688768 (4.70 μM), also exhibited LSG activity but not ABS activity, suggesting that these compounds are likely targeting cellular proteins and pathways that are essential in the gametocyte stage and not in the asexual stage. It must be noted that this is only a single determination of the IC_50_; however, compounds that display LSG activity represent suitable starting points for developing transmission blocking antimalarial agents.

Compounds with activity against the P. falciparum parasite from the Discovery Biology data may be expected to also display activity against either Cryptosporidium or Toxoplasma spp., since they are all from the *Apicomplexa* phylum. However, of the 24 compounds in the PBox described as targeting Toxoplasma or Cryptosporidium, only 2, MMV688703, and MMV675968, possessed antiplasmodial activity. The mechanism of action is known for both the *Apicomplexa* active compounds. MMV675968 is known to target Cryptosporidium dihydrofolate reductase (DHFR) (80) and is structurally similar to antiplasmodials that are known to target DHFR (81), and it is therefore likely that MMV675968 targets Plasmodium DHFR. MMV688703 is known to be a potent inhibitor of Toxoplasma cGMP-dependent protein kinase, involved in the regulation of calcium critical for signaling in Toxoplasma (82). It has also been shown that MMV688703 targets the Plasmodium cGMP-dependent protein kinase (PfPKG) and that this kinase plays an important role in asexual-stage parasite and gametocyte development (83, 84). Although this compound potently inhibits PfPKG (IC_50_ = 8.5 nM), it has a modest IC_50_ in a low micromolar range against the ABS P. falciparum parasite, a finding consistent with the IC_50_ determined here (3.16 μM). The function of PfPKG has been shown to be important in early gametocytogenesis (85) but not late-stage gametocytogenesis, corroborating the data here to suggest that MMV688703 does not affect this stage of gametocytogenesis.

### Potential chemical starting points for kinetoplastid drug discovery programs from the PBox “kinetoplastid disease indication” compound set.

Of the 70 compounds within the kinetoplastid compound set, a number interesting as potential starting points for hit-to-lead and target identification purposes. Seventy percent of the kinetoplastid set of compounds were active against one or more of the three kinetoplastid pathogens tested in this study. Below, we classified the compounds into selective (based on an MMV selection index applied to compounds contained within PBox of >5-fold) individual parasite activities and those which are active across more than one parasite. An SI of 5 is lower than generally accepted by this laboratory as a starting point for chemistry optimization; however, for normalization with the PBox compound selection criteria we have also used 5-fold as a measure of selectivity. Greater selectivity for mammalian cells (at least 10-fold for kinetoplasts and 50-fold for malaria) is desirable for compound progression.

### *T. brucei brucei* and T. cruzi.

MMV688550, an imidazo[1,2]purine, displayed good selectivity for both *T. brucei brucei* and T. cruzi, with SIs of >154 and >31, respectively, compared to HEK293 cell growth inhibition and represents a suitable starting point for targeting both *T. brucei brucei* and T. cruzi parasites. MMV688372, a substituted 2-phenylimidazopyridine, showed SIs of 24 for T. cruzi and 230 for *T. brucei brucei* in relation to HEK293 cells. Although MMV688372 demonstrated an IC_50_ of 1.15 μM against L. donovani, the selectivity over HEK293 cytotoxicity was not >5-fold. MMV688372 was identified through the development of an oxazolopyridine series and was shown to possess potent activity against *T. brucei brucei*, with an example from this series demonstrating efficacy in a mouse model of trypanosomiasis (86). A separate study also found representatives from the related oxazol-2-ylanilide to be active against *T. brucei brucei* (33), and in a metabolomics study an analogue was discovered to target sphingolipid metabolism in *T. brucei brucei* (87). In addition, a structurally related azabenzoxazole has recently been identified as a proteasome inhibitor in all three kinetoplastid parasites as a part of the Novartis Research Foundation Genomics Institute kinetoplastid screening campaign (88).

MMV688797, MMV688958, and MMV688795 are all structurally related to the 2-aryl oxazole chemotype, which is reported to display activity against T. brucei rhodesiense (89), and activity was identified against *T. brucei brucei* and T. cruzi from a hit-to-lead program targeting *T. brucei brucei* (33). Although the antikinetoplastid activity of these compounds is not novel, the target is unknown and remains to be elucidated. MMV688467 is a butyl sulfanilamide and a known inhibitor of microtubule formation in *T. brucei brucei* (90). MMV688467 also demonstrated modest activity against T. cruzi (IC_50_ of 3.98 μM). MMV689028, a benzyl piperazine, originated from the GSK Kinetoplastid Screening program (91) and exhibited potent activity against both *T. brucei brucei* (Discovery Biology, IC_50_ of 0.24 μM; PBox, 0.14 of μM) and T. cruzi (Discovery Biology, IC_50_ of 0.67 μM; PBox, IC_50_ of 1.20 μM). MMV689028 provides a suitable starting point for target identification studies for *T. brucei brucei* and T. cruzi.

MMV688796 (a 2,4-substituted furan), MMV689028 and MMV689029 (both benzyl piperazines), and MMV688371 (a benzamide) present suitable starting points for both *T. brucei brucei* and T. cruzi. All of these compounds also demonstrated activity against the HAT infective species T. brucei rhodesiense, supporting the potential for these compounds as novel and attractive targets for further development.

### L. donovani and T. cruzi.

MMV690102, MMV690103, and MMV689437, which belong to the pyrimido[4,5-d]pyrimidine-2,4,7-triamine chemotype, all demonstrated activity against L. donovani and T. cruzi, with no activity displayed in the HEK293 assay at the concentrations tested. All of the compounds were previously identified from the GSK kinetoplastid screen. Structurally related compounds have been found to be inhibitors of DHFR isolated from mammals (81), which is potentially the target in these parasites. It should also be noted that these compounds are not active against malaria or *T. brucei brucei*, for which DHFR is a recognized drug target. However, MMV675968 (cryptosporidiosis set), a structurally related diaminoquinazoline, has potent activity against the asexual stage of P. falciparum (3D7 IC_50_, 0.03 μM; SI HEK, 7.4; SI HepG2, 113), and DHFR has been shown to be the cellular target (92). This suggests a structural divergence between DHFR orthologues expressed by these species of parasites. MMV690102, MMV690103, and MMV68943 are considered promising starting points for hit-to-lead optimization; however, their activity against the L. donovani and T. cruzi DHFR remains to be confirmed.

For many of the compounds displaying activity against kinetoplastid parasites, there was a lack of activity against L. donovani intracellular amastigotes. This could be associated with environmental factors within the acidic vacuole, where the parasite resides (93), or indicate that division of the intracellular form is slow (94). In contrast to *T. brucei brucei*, there is also an additional host membrane which the compounds must cross to have affect the Leishmania parasite. Previous studies have shown a poor correlation of compound activity against axenic amastigotes and intracellular amastigotes (94). Data were provided for a selection of compounds from the PBox tested against L. donovani axenic amastigotes; however, many other compounds were not tested. Further evaluation of PBox compounds against axenic forms of L. donovani and also the promastigote forms could provide insights into the differences observed between the kinetoplastids and expand our understanding of compound action against the intracellular form of the parasite.

### Activity against *T. brucei brucei* and L. donovani.

MMV688271 and MMV688179 are bisaryl guanidinium analogues that have been identified with activity against L. donovani, T. cruzi (77), and *T. brucei brucei*. DNA affinity of guanidinium-like derivatives has been reported in parasites as the potential cellular target (78), and therefore the mammalian selectivity of this compound class should be monitored due to the potential mode of action.

### Activity against T. cruzi only.

Of seven compounds demonstrating activity against T. cruzi alone, MMV688942, MMV688415, MMV688943, MMV689244, MMV689243, and MMV676048 had a subefficacious effect (less than 100% *E*_max_ as demonstrated by posaconazole from Table 2) in the Discovery Biology assay in that they did not clear 100% of the parasite from host cells. This property has been associated with the activity of azole antifungals and T. cruzi cytochrome P450 inhibitors (T. cruzi CYP51) against the parasite in the Discovery Biology image-based assay (31) and also two other assay formats reported in the literature (95, 96). These compounds are therefore likely to be associated with CYP51 or sterol biosynthesis activity, and several have been confirmed in the literature, such as, for example, Bitertanol (MMV688942) or difenoconazole (MMV688943), both azole antifungals targeting CYP51 in common fungal pathogens. Considering the recent failure rate of CYP51 inhibitors against T. cruzi in preclinical and clinical trials (97), these are not considered attractive starting points for targeting T. cruzi. MMV689709, a 3-substituted indazole, is a suitable starting point, with a modest IC_50_ of 3.32 μM.

### Activity against *T. brucei brucei* only.

Three-quarters of the compounds active in the kinetoplastid set were *T. brucei brucei* active, with 54% demonstrating activity only against *T. brucei brucei*. Of the 18 compounds that demonstrate activity against *T. brucei brucei* specifically, 16 were selective for the parasite, whereas two (MMV595321 and MMV67660) demonstrated comparable activity against mammalian HEK293 cells.

MMV690028 and MMV690027, both hexahydrophthalazinones, have reported activity against trypanosomal phosphodiesterase (PDE) B1 (98). MMV690027 has been shown to inhibit human PDE4 and has been repurposed as a potent inhibitor of *T. brucei brucei* PDE B1 (99). The activity data for MMV690028 against L. donovani and for MMV690027 against T. cruzi and L. donovani has not been reported previously.

MMV202553 and MMV688793, both 2-pyridyl benzamides, exhibited modest activity against *T. brucei brucei* (IC_50_s of 1.0 and 1.2 μM, respectively); however, they are likely to be difficult to optimize since this chemotype is commonly found in serine protease factor Xa inhibitors (100). MMV188296, a 2-indolinecarboxamide, was previously optimized to potently inhibit and selectively target *T. brucei brucei* (101). MMV688776, a pyrazoloquinazoline, previously considered for treatment of mycobacteria but not trypanosomes (102), is potentially a suitable starting point for optimization against HAT, due to its demonstrated activity against the human infective form.

### Potential chemical starting points for malaria drug discovery programs from the PBox antiplasmodial compound set.

Extensive screening campaigns for the identification of antiplasmodial compounds have been completed over the past 5 to 10 years (35, 54, 55), with reports of between 4 and 12 million compounds tested thus far, reducing, but not completely negating, the likelihood of identification of new antiplasmodial compound classes. Compounds within the malaria set constitute one-third of the PBox, with approximately 100 ABS active compounds with no known mechanism of action. Compounds that exhibit antiplasmodial activity with an unknown mechanism of action not only offer a suitable starting point for medicinal chemistry optimization but also offer the potential to be used as a chemical probe to reveal previous unstudied targets and mechanisms essential for Plasmodium parasite survival. Future testing of PBox against some of the malaria target and pathway specific assays (16, 40, 103–111) available throughout the global research community will highlight potential targets for these orphan compounds. Classes with novel mechanisms of action are in dire demand in order to attempt to combat resistance generation to numerous small-molecule agents that have the same mechanism of action.

There are 13 compounds from the malaria set with activity across ABS, gametocytes, and the liver stage. These compounds are of high priority for malaria drug discovery programs. MMV634140 and MMV667494, quinolone 4-carboxamides, have recently been identified as inhibitors of the Plasmodium translational elongation factor 2 (elF2) (42), while MMV010576 ((2-amino pyridine), the “hit” molecule (112) from which the antimalarial drug candidate MMV390048 arose, and MMV085499 (2-amino pyrazine) (113) are expected to have phosphatidylinositol 4-kinase (PI4K) activity based on the elucidated target for clinical candidates MMV390048 (114) and MMV642943 (drug candidate UCT943). MMV024443 (an indole-2-carboxamide) has been shown to target CDPK1 from Plasmodium parasites (115), along with MMV023985 and MMV010545, for which CDPK1 or PK7 have been proposed as the target (114, 116–120). The compounds above with PI4K, elF2, CDPK1, or PK7 targets in Plasmodium parasites have shown no activity in the kinetoplast assays undertaken by Discovery Biology.

The remaining six compounds (MMV026356, MMV019189, MMV023860, MMV019721, MMV022029, and MMV024035) do not have any associated target reported in the literature and, although they are only moderately active (ABS, 0.2 to 7 μM), they are therefore considered to be very interesting starting points for malaria drug discovery. There are also eight compounds (MMV023233, MMV020081, MMV022478, MMV062221, MMV108852, MMV560185, MMV1028806, and MMV1030799) that demonstrate activity against stage V gametocytes (and stage IV as determined here); these compounds are either active or inactive against the ABS and provide potential hits for malaria transmission blocking strategies.

This is the first comprehensive report to canvas the activity of the PBox compound collection against kinetoplastid and malaria parasites with a thorough analysis of the novelty of hits and the potential for either further biological characterization or target identification in the drug discovery arena. The simultaneous screening of the PBox against these parasite species has resulted in the identification of new and novel compounds, which offer potential starting points in early drug discovery campaigns. The outcome from this investigation will allow researchers from around the world to prioritize compounds or complement their studies based upon the information provided here. The data provided in this study support the MMV open-access initiative to build upon the knowledge that is publicly available for these compounds, with our data to be shared in ChEMBL.

## MATERIALS AND METHODS

### Pathogen Box collection.

From the Pathogen Box website (http://www.pathogenbox.org/) a link to a supporting information Excel file that contains the following information is provided. The compound plate layout, which compounds are in each “pathogen set,” the chemical structure and formula, SMILE string, salt, molecular weight, cLogP, and the confirmed biological activity against the respective pathogen set are included. HepG2 cytotoxicity data (CC_20_ or CC_50_) are also provided for approximately three-quarters of the compounds. Acknowledgments to the testing platforms for confirmation of biological activity are provided; however, no details of the assay specifics are recorded.

### Compound handling.

The PBox was received as five 96-well plates containing 10 μl of 10 mM stocks of 400 compounds. Compounds Australia processed the compounds by the addition of 10 μl of DMSO to all compound-containing wells to give a total volume of 20 μl, which was then transferred and compressed to make two identical sets of low-dispense-volume plates (384-well), each containing 10 μl of compounds in a 5 mM DMSO stock solution.

**FIG 3:**
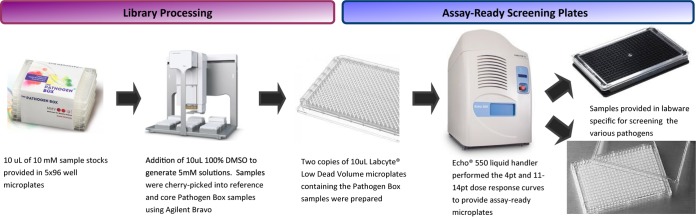
Compound and plate processing of PBox by Compounds Australia.

Australian biosecurity level 2 (BC2) and physical containment level 2 (PC2) restrictions associated with working with these specific pathogens and different assay formats dictated the preparation of the assay plates. In the case of P. falciparum, assay-ready plates were prepared with nanoliter acoustic dispensing directly into imaging plates. For all other assays involving adherent infected cell lines, nanoliter to microliter acoustic dispensing was performed in sterile intermediate plates (Fig. 3). All compound plates were then transferred to the appropriate assay platform in a BC2/PC2 facility.

### Assays.

The PBox was tested in P. falciparum (*Pf*3D7 was obtained from BEI Resources Manassas, VA, and *Pf*NF54^-pfs16-LUC-GFP^ was kindly supplied by David Fidock, Columbia University, New York, NY) ABS and LSG assays. Also tested were the kinetoplastid pathogens T. cruzi (kindly provided by Frederick Buckner, University of Washington, Seattle, WA), L. donovani (American Type Culture Collection [ATCC], Manassas, VA), and *T. brucei brucei* (kindly provided by Achim Schnaufer, University of Edinburgh, Edinburgh, Scotland), and a resazurin metabolic viability assay was tested for mammalian cell cytotoxicity utilizing HEK293 cells (ATCC). (All published assays are briefly described in Supplemental File 2.)

### Leishmania donovani intracellular amastigote assay. (i) Cell culture.

L. donovani MHOM/IN/80/DD8 (ATCC 50212) promastigote parasites were maintained in modified M199 Hanks salt medium (pH 6.8) supplemented with 10% heat-inactivated fetal bovine serum (FBS) at 27°C. Parasites were subcultured every 7 days at a concentration of 10^5^ cells/ml. THP-1 (ATCC TIB202) cells were maintained in RPMI medium and 10% FBS at 37°C/5% CO_2_. The cells were subcultured every 2 to 3 days in order to maintain a cell density between 2 × 10^5^ and 1 × 10^6^ cells/ml.

### (ii) Assay protocol.

THP-1 cells were seeded in 384-well cell carrier imaging plates (Perkin-Elmer, Waltham, MA) using a Bravo automated liquid handling platform (Agilent Technologies, Santa Clara, CA) at a concentration of 12,500 cells per well in RMPI plus 10% fetal calf serum (FCS) medium containing 25 ng of phorbol 12-myristate 13-acetate (PMA)/ml in order to induce differentiation of the THP-1 cells. Assay plates were incubated at room temperature for 30 min to allow cells to adhere before being incubator at 37°C and 5% CO_2_ for 24 h. After incubation, the PMA was removed by discarding the medium within wells and washing the assay plates three times in phosphate-buffered saline (PBS) on an EL405 plate washer (Biotek Instruments, Winooski, VT). After washing, 40 μl of fresh RMPI and 10% FBS medium were added to the assay plates, followed by incubation for 48 h at 37°C and 5% CO_2_.

The number of metacyclic promastigotes present in a 7-day-old L. donovani DD8 promastigote culture was subsequently determined, and parasites were added to the assay plates containing the transformed THP-1 cells (72 h after initial cell seeding) at a multiplicity of infection of 1:5 (ratio of host cells to parasites). Assay plates were incubated at room temperature for 30 min, followed by 24 h incubation at 37°C and 5% CO_2_. Noninternalized parasites were subsequently removed by aspirating the medium within wells and washing the assay plates six times in PBS on a EL405 plate washer before the addition of 45 μl of fresh RMPI (10% FBS and 25 ng/ml PMA). Controls consisted of positive wells containing a final assay concentration of 2 μM amphotericin B, and negative wells containing 0.4% DMSO were used as in-plate controls for all experiments. Then, 1 μl of each compound, prepared by Compounds Australia, was diluted by the addition of 24 μl of RPMI medium containing no FCS. Next, 5-μl portions of this dilution were dispensed via a Bravo liquid handler to assay plates to give final assay concentrations of 20, 10, 5, and 0.5 μM (at 0.4% DMSO). For IC_50_ confirmation, the final assay concentrations ranged from 20 to 0.001 μM. Assay plates were incubated for 96 h at 37°C and 5% CO_2_ before being fixed with 4% paraformaldehyde and stained with SYBR green and CellMask Deep Red (ThermoFisher Scientific, Wlatham, MA).

Images were acquired on an Opera high-content imaging system (Perkin-Elmer). Healthy host (THP-1) cells were identified based on the CellMask Deep Red cytoplasmic and SYBR green nuclear area and intensities. Segmentation of nuclear and cell boundaries was used to identify the region of host cell cytoplasm. Intracellular parasites were then identified within this region based on spot detection algorithms of the SYBR green staining (with size and intensity measurements used to define parasite nucleus of kinetoplast) to determine the number of parasites present within THP-1 host cells. An infected cell was defined as a host cell containing >3 parasites within the cytoplasm boundary. The compound activity was determined based on the number of infected cells normalized to the positive (2 μM amphotericin B) and negative (0.4% DMSO) controls. Nonlinear sigmoidal dose-response curves with no constraints were plotted, and IC_50_s were calculated using GraphPad Prism 6. The IC_50_s were calculated from two independent experiments.

### Pathogen Box primary screening.

The PBox was initially tested at four doses (20, 10, 5, and 0.5 μM). Active compounds taken for IC_50_ determination (i.e., the concentration at which 50% of the parasites were killed) were selected individually for each of the parasite assays performed based on the level of activity, correlation with data presented within the PBox supporting information, and whether the observed activity was novel to a pathogen other than that already determined within PBox supporting information file.

### IC_50_ determination. (i) Kinetoplastids.

Compounds demonstrating > 50% inhibition at 10 μM for L. donovani and T. cruzi were progressed for IC_50_ evaluation, while all compounds demonstrating >50% activity at 5 μM for *T. brucei brucei* were taken for IC_50_ determination.

### (ii) Plasmodium.

Compounds with activity greater than 60% at 5 μM observed for either ABS or LSG assays were considered for IC_50_ determination. Compounds from the subset of the PBox designated “malaria disease indication” had IC_50_s determined only if there were discrepancies with the data provided within the PBox supporting information file or if LSG activity was observed. All compounds from “other pathogen disease indication” compound subsets with activity greater than 60% for ABS or LSG at 5 μM underwent IC_50_ determination. Simultaneous testing of compounds in ABS, LSG, and HEK293 cytotoxicity assays were performed.

## Supplementary Material

Supplemental material
