# Supplementary material for: Screening the Medicines for Malaria Venture Pathogen Box across Multiple Pathogens Reclassifies Starting Points for Open-Source Drug Discovery
Source: Antimicrob Agents Chemother. 2017 Aug 24;61(9):e00379-17. doi: 10.1128/AAC.00379-17 (PMC5571359; doi:10.1128/AAC.00379-17)
Supplement: Supplemental material [file supp_61_9_e00379-17__index.html]

Supplemental material 

# Screening the Medicines for Malaria Venture Pathogen Box across Multiple Pathogens Reclassifies Starting Points for Open-Source Drug Discovery

## Supplemental material

- Supplemental file 1 -

  Data Set S1

  XLSX, 462K
- Supplemental file 2 -

  Figure of cell destruction and detailed assay methods

  PDF, 301K
