# Supplementary material for: Screening the Medicines for Malaria Venture Pathogen Box across Multiple Pathogens Reclassifies Starting Points for Open-Source Drug Discovery
Source: Antimicrob Agents Chemother. 2017 Aug 24;61(9):e00379-17. doi: 10.1128/AAC.00379-17 (PMC5571359; doi:10.1128/AAC.00379-17)
Supplement: Supplemental material [file AAC.00379-17_zac009176504s2.pdf]

Screening the MMV Pathogen Box across multiple pathogens reclassifies starting points for open source drug discovery.

Supporting information 2

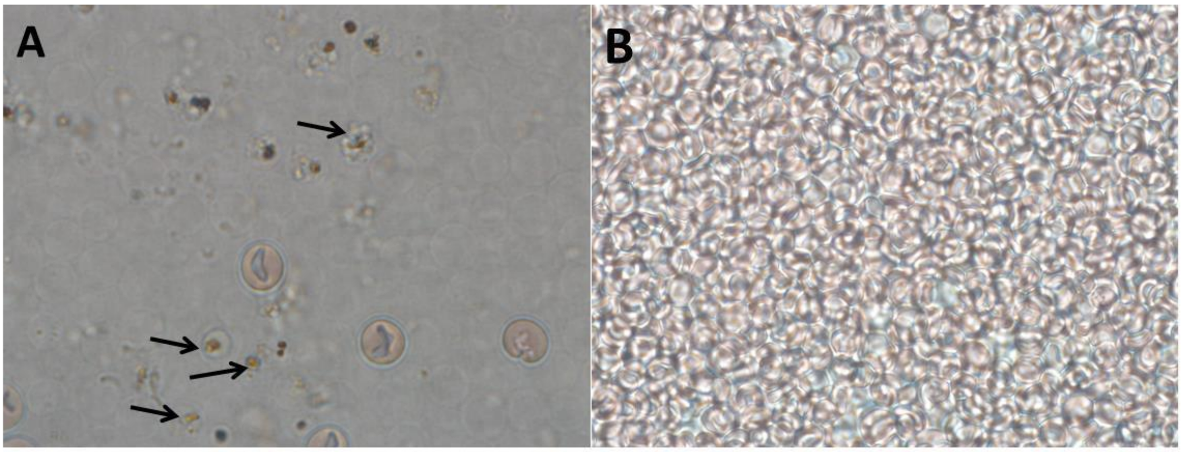

A Compounds with strange shinny/glassy effect

B Artemisinin treated RBC's which look the same as untreated RBC's

Screening the MMV Pathogen Box across multiple pathogens reclassifies starting points for open source drug discovery.

Sandra Duffy[1], Melissa L Sykes[1], Amy J Jones[1], Todd B Shelper[1], Moana Simpson[2], Rebecca Lang[2], Sally-Ann Poulsen[2], Brad E Sleebs[3,4], Vicky M Avery[1,2]

[1] Discovery Biology, Griffith Institute for Drug Discovery, Griffith University, Nathan, Queensland, 4111, Australia

[2] Compounds Australia, Griffith University, Nathan, Queensland, 4111, Australia.

[3] The Walter and Eliza Hall Institute of Medical Research, Parkville Victoria, 3052, Australia

[4] Department of Medical Biology, The University of Melbourne, Parkville, Victoria, 3010, Australia

Supporting information 3

Plasmodium falciparum asexual blood stage assay (ABS).

The asexual blood stage HCS image based assay utilises the DNA-intercalating dye DAPI (4', 6',-diamidino-2-phenylindole) to monitor changes in the parasite number observed within infected erythrocytes [1].

Briefly, *Plasmodium falciparum* parasites (3D7, Chloroquine sensitive strain) were cultured in RPMI1640 media supplemented with hypoxanthine, Hepes, 2.5 mg / ml AlbuMAX® II and 5% human serum.

Compounds were incubated in the presence of 2% sorbitol synchronized ring stage parasites in a total assay volume of 50 μL, for 72 hrs at $37^{o}C$ and 5% CO_2, 5% O_2 in poly-*D*-lysine coated imaging plates. After incubation, the plates were stained with DAPI in the presence of saponin and Triton X-100 and incubated for a further 8 hrs at room temperature in the dark before imaging using the Opera™ (PerkinElmer). The digital images obtained from each well were analysed using spot detection software

(Acapella) where fluorescent spots which fulfilled the criteria established for a stained parasite were counted. The percent inhibition of parasite replication was calculated using 0.4% DMSO (0% inhibition) and 5 µM Puromycin (100% inhibition) to normalize all the activity data. IC_{50} values were calculated using GraphPad Prism 4.

Plasmodium falciparum late stage gametocyte assay (LSG)

The sexual blood stage HCS image based assay utilizes the transgenic parasite, NF54$^{-pfs16-LUC-GFP}$ which expresses GFP under the control of the pfs16 promoter. The GFP is detectable throughout the gametocyte developmental path to that of a mature stage gametocyte. In combination with the mitochondrial viability marker, MitoTracker® Red CM-H2Xros, viable mature stage gametocytes are identified [2].

In brief, NF54$^{-pfs16-LUC-GFP}$ parasites were cultured in RPMI1640 media supplemented with hypoxanthine, Hepes, 2.5 mg / ml AlbuMAX® II, 5% human serum plus blasticidin for selection purposes. The parasites were processed through a synchronous single cycle of “stress” to induce gametocytogenesis. Under N-Acetyl-glucosamine (NAG) treatment asexual parasites were removed from the developing gametocyte culture. Resultant stage IV gametocytes were isolated by VarioMACS™, separator columns, adjusted to 10% gametocytes and added to poly-*D*-lysine coated imaging plates which contained the compounds of interest. After 72 hrs of incubation at 37°C and 5% CO_2, 5% O_2, MitoTracker® Red CM-H2Xros was added to all wells and the plates incubated for a further 16 hrs . The plates were then imaged using an Opera® High Content Screening System to identify elongated gametocytes with demonstrated mitochondrial viability. The percent inhibition of gametocyte inhibition was calculated using 0.4% DMSO (0% inhibition) and 5 µM Puromycin (100% inhibition) to normalize all the

activity data. IC_{50} values were calculated using Graphpad Prism 4. Puromycin, chloroquine, artesunate, DHA, pyronaridine and pyrimethamine were also tested in IC_{50} format to act as internal reference compounds.

Intracellular anti-Trypanosoma cruzi Assay Protocol

The host cell cytoplasm, in which the parasite resides, is identified by the fluorescent marker, HCS CellMask Green. Parasite and host cell nuclei are detected with the fluorescent DNA intercalator, Hoechst 33342. The Opera® High Content Screening System is used to take images of wells and a spot detection algorithm is used to identify parasites within the cytoplasm [3].

Briefly, 1x 10^3 3T3 cells were added to collagen coated 384-well plates and following 24 hrs incubation at 37°C, a multiplicity of infection (MOI) of 5:1 parasite: host cells were added to the plate using a Multidrop liquid handler. Following additional 24 hrs incubation, extracellular parasites were washed from the plates and RPMI with no phenol red, supplemented with 10% FCS and 100 IU / mL penicillin/streptomycin was added to all wells. The final volume of media in the primary screening assay, before addition of compound, was modified slightly to 55 µL. One µL of compounds, provided by Compounds Australia, were diluted by the addition of 25 µL of sterile water. This volume was modified from addition of 20 µL of water, as these compounds were processed as the other assays undertaken (*P. falciparum*, *T. b. brucei* and *L. donovani*). Five microliters of this dilution was added to assay plates. The final concentrations of compound in the assay were 16.7, 8.3, 4.2 and 0.42 µM. For retest, twenty microliters of water was added to 1 µL of compounds in DMSO, then 5 uL was

added to an assay volume of 60 µL to maximise the compound concentration and enable identification of IC_{50} values. The final concentrations of compounds in the assay were from 19.8 µM to 9.9e-4 µM. Following 48 hrs incubation in the presence of compounds, wells were fixed, stained and read on the Opera® High Content Screening System to assess compound activity.

The Z′-factor, as a measure of assay reproducibility, was determined from a plate containing a third each of a final concentration of 0.37% DMSO, nifurtimox (NFX) at 12 µM and puromycin at 30 µM. The positive control used to calculate activity against host cells was 30 µM of puromycin, whilst for the amastigotes was 12 µM of NFX. To assess the sensitivity of the *T. cruzi* assay, activity of the reference compounds benznidazole, NFX, posaconazole and puromycin were determined.

T. b. brucei Resazurin Viability Assay

The assay utilises *T. b. brucei* 427 bloodstream trypanosomes and the redox-indicator resazurin as a measure of parasite viability [4].

T. b. brucei trypanosomes were cultured *in vitro* in HM1-9 medium supplemented with 10% foetal bovine serum (FCS) at 37°C in 5% CO_2. Fifty-five µL of logarithmic phase *T. b. brucei* parasites (1200 cells/ml) were added to 384-well microtiter plates and incubated for 24 hrs at 37°C /5% CO_2. Compounds were subsequently added to the parasites in a volume of 5 µL to give a final assay volume of 60 µL. Following incubation for 48 hrs at 37°C / 5% CO_2, 10 µL of 0.49 mM resazurin prepared in HMI-9 media +10% FCS was added to assay plates and incubated for a further 2 hrs at 37°C / 5% CO_2 followed by 22 hrs at room temperature. Assay plates were read at 535 nm excitation / 590 nm emission on an Envision® micro-plate reader. The percent

inhibition of parasite replication was calculated using 0.4% DMSO (0% inhibition) and 5 µM Puromycin (100% inhibition) to normalize the activity data. IC_{50} values were subsequently calculated using the software GraphPad Prism 6. The reference compounds pentamidine and diminazine aceturate served as assay controls.

HEK293 resazurin cell cytotoxicity assay

The assay utilises HEK293 mammalian cells and the redox-indicator resazurin as a measure of parasite viability.

HEK293 cells (2000 cells per well) were seeded in 384 well micro-titre plates in 45 µL of DMEM supplemented with 10% FCS. Compounds were prepared in 100% DMSO and serially diluted to give 16 point dose response curves (DRC) . The compound plates were subsequently diluted 1:25 in sterile water and 5 µL of this dilution added to assay plates. Assay plates were incubated for 72 hrs at 37°C / 5% CO_2. The culture media was removed from the plates and 40 µL of 40 µM resazurin in DMEM without FCS added to all wells. The plates were incubated for a further 6 hrs then fluorescent intensity measured at 535 nm excitation / 590 nm emission on an Envision® micro-plate reader. Puromycin (5 µM) and 0.4% DMSO were included in each assay plate as positive and negative controls, respectively. IC_{50} values were subsequently calculated using the software GraphPad Prism 6.

References

1. Duffy S, Avery VM. Development and optimization of a novel 384-well anti-malarial imaging assay validated for high-throughput screening. The American

journal of tropical medicine and hygiene. 2012;86(1):84-92. doi: 10.4269/ajtmh.2012.11-0302. PubMed PMID: 22232455; PubMed Central PMCID: PMC3247113.

2. Duffy S, Avery VM. Identification of inhibitors of *Plasmodium falciparum* gametocyte development. Malaria Journal. 2013;12(1):408. doi: 10.1186/1475-2875-12-408.

3. Sykes ML, Avery VM. Development and application of a sensitive, phenotypic, high-throughput image-based assay to identify compound activity against *Trypanosoma cruzi* amastigotes. Int J Parasitol Drugs Drug Resist. 2015;5(3):215-28. doi: 10.1016/j.ijpddr.2015.10.001. PubMed PMID: 27120069; PubMed Central PMCID: PMC4847003.

4. Sykes ML, Avery VM. Development of an Alamar Blue viability assay in 384-well format for high throughput whole cell screening of *Trypanosoma brucei brucei* bloodstream form strain 427. The American journal of tropical medicine and hygiene. 2009;81(4):665-74. doi: 10.4269/ajtmh.2009.09-0015. PubMed PMID: 19815884.
